# Gestational Intermittent Hypoxia Impairs AT_2_R-Mediated Vascular Protection in Female Offspring on a High-Fat, High-Sucrose Diet

**DOI:** 10.26502/jbb.2642-91280150

**Published:** 2024-06-14

**Authors:** Ruolin Song, Pankaj Yadav, Jay S Mishra, Sri Vidya Dangudubiyyam, Sathish Kumar

**Affiliations:** 1 Department of Comparative Biosciences, School of Veterinary Medicine, University of Wisconsin, Madison, WI 53706, USA; 2 Department of Obstetrics and Gynecology, School of Medicine and Public Health, University of Wisconsin, Madison, WI 53792, USA

**Keywords:** Intermittent hypoxia, Offspring, high-fat diet, AT_2_R, Blood pressure, Endothelium

## Abstract

**Background:**

Gestational intermittent hypoxia (GIH), a hallmark of maternal obstructive sleep apnea, sex-differentially causes hypertension and endothelial dysfunction in adult male offspring but not in females. This study investigated whether the GIH-exposed female offspring, a “protected” group against the hypertensive effects of maternal GIH exposure, exhibit increased susceptibility to hypertension and cardiovascular dysfunction when fed a high-fat high-sucrose (HFHS) diet and whether this effect could be reversed by pharmacological intervention activating the angiotensin II type 2 receptor (AT_2_R).

**Methods:**

Female offspring of control and GIH-exposed (10.5% O_2_, 8 h/d, E10–21) dams were assigned either an HFHS diet or a standard diet from 12 weeks of age. Blood pressure was monitored. At 28 weeks, a systemic CGP42112 (AT_2_R agonist) or saline infusion was administered through the osmotic pump. At 30 weeks, the heart was weighed and collected for H&E staining, mesenteric arteries for vascular reactivity assessment and protein analysis, and plasma for ELISA.

**Results:**

The HFHS diet induced similar increases in body weight gain and blood pressure in control and GIH female offspring. HFHS feeding did not affect heart structure, but impaired endothelial-dependent vascular relaxation with associated decreased AT_2_R and eNOS expression and reduced plasma bradykinin levels in both control and GIH offspring. CGP42112 administration effectively mitigated HFHS-induced hypertension and endothelial dysfunction only in control offspring, accompanied by restored AT_2_R, eNOS, and bradykinin levels, but not in the GIH counterparts.

**Conclusion:**

These findings suggest that GIH induces endothelial dysfunction and AT_2_R insensitivity in female offspring exposed to an HFHS diet.

## Introduction

Maternal in-utero conditions during pregnancy are intricately associated with the health trajectories of their offspring [1]. Among all factors affecting in-utero conditions, maternal Obstructive Sleep Apnea (OSA), a sleep-disordered breathing characterized by repeated episodes of respiratory arrest, has gained increasing attention due to its prevalence in pregnant women – up to 26% in the third trimester [2–4], and its association with adverse fetal outcomes [5–7]. Previous animal studies suggest that gestational intermittent hypoxia (GIH) exposure, a hallmark of OSA, induces sex-specific programming of reduced vascular relaxation in the offspring. Specifically, in pre-pubertal offspring, GIH leads to elevated blood pressure (BP), left ventricle hypertrophy, and cardiac dysfunction in both sexes, but more profoundly in males [8]. However, in adult offspring, GIH-induced increases in BP are observed exclusively in males with associated endothelial dysfunction and sex steroid hormone imbalances, whereas female GIH offspring stabilize their BP after puberty [9, 10]. This restoration of normotensive BP in post-pubertal female GIH offspring is attributed to a protective role of functional ovaries [11]. However, questions persist regarding whether these GIH-exposed female offspring, when exposed to additional environmental hypertensive stressors, exhibit increased susceptibility to hypertension and whether this could be reversed by pharmacological intervention activating the angiotensin II type 2 receptor (AT_2_R).

High-fat, high-sucrose (HFHS) diets are major contributors to cardiovascular disease. Multiple mechanisms, including the renin-angiotensin system (RAS) [12], chronic inflammation [13], baroreflex function [14], and sympathetic nerve activity [15], have been implicated in the pathogenesis of HFHS-induced hypertension. The RAS is particularly important, as it links BP regulation, inflammation, and glucose/lipid metabolism [16, 17]. Angiotensin II (Ang II) is the primary effector of the RAS and exerts its effects through two main receptors, angiotensin II type 1 receptor (AT_1_R) and AT_2_R. AT_1_R activation evokes vasoconstriction in the smooth muscle cells [18]. AT_2_R signaling, on the other hand, is antagonistic to AT_1_R signaling and promotes vasodilation via nitric oxide (NO) and bradykinin system [19].

HFHS diets disrupt AT_1_R/AT_2_R balance by upregulating AT_1_R expression in vascular tissues and downregulating AT_2_R expression [20–22]. The combined consequence is an overall shift toward Ang II’s pro-hypertensive and pro-inflammatory effects, contributing to the development of hypertension and vascular dysfunction. RAS interventions can restore BP and endothelial dysfunction in hypertension. For example, combined AT_1_R and angiotensin-converting enzyme (ACE) blockade has potent antihypertensive effects in the spontaneously hypertensive rat (SHR) model [23], possibly by directing Ang II toward the vasodilatory AT_2_R signaling and increasing bradykinin bioavailability. Similarly, the AT_2_R agonist Compound 21 mitigates vascular dysfunction and hypertension in gestational hypertension [24].

The present study aimed to evaluate the impacts of an HFHS diet as a “second hit” on the adult female offspring born to GIH-exposed dams, a group previously found to be “protective” against maternal GIH-induced cardiovascular dysfunctions, and to investigate whether stimulating AT_2_R function can reverse the effects of HFHS diet in these offspring. To this end, an HFHS diet and AT_2_R agonist CGP42112 were administered to GIH-exposed female offspring rats. BP and vascular function were assessed. Factors regulating vascular tone in plasma and their receptor levels in mesenteric arteries were measured.

## Materials and Methods

All animal procedures were conducted according to the Guide for the Care and Use of Laboratory Animals (National Institutes of Health Publication No. 85–23, revised 1996) and approved by the Institutional Animal Care and Use Committee (IACUC) at the University of Wisconsin-Madison under protocol V005847.

### Intermittent Hypoxia Exposures during Pregnancy

Timed-pregnant Sprague-Dawley (SD) rats were obtained from Charles River Laboratories (Wilmington, MA, USA) at gestational day (GD) 8 (plug detection designated as GD1) and housed in a controlled environment with a 12:12 light-dark cycle, providing *ad libitum* access to food and water. From GD10 to GD21, dams designated for the GIH group were exposed to an intermittent hypoxia protocol mimicking human OSA [25, 26]. This protocol consisted of eight-hour daily cycles (during the presumed sleep period) of alternating 2-minute hypoxic (10.5% O_2_) and normoxic (21% O_2_) episodes. The chosen parameters aimed to replicate human desaturation and reoxygenation patterns observed in OSA patients [25, 26]. The GD10-GD21 exposure window targets mid-to-late gestation, a clinically relevant period as OSA prevalence in pregnant women significantly increases during late pregnancy (26.7% vs. 10.5% in the third vs. first trimester, respectively) [27]. Control group dams received identically timed exposures to room air with gas flow rates matching those of the GIH group. All hypoxia exposures ceased one day prior to parturition to prevent direct pup exposure to hypoxic episodes.

### Post-Weaning Diet for the Offspring

Pups were weaned at postnatal day 21 and reared in separate cages based on their sex. This study focused exclusively on female offspring. All pups received standard laboratory chow (2020X, Envigo, Indianapolis, IN, USA) *ad libitum* until postnatal day 84 (12 weeks of age). At this time, offspring were randomly assigned to one of two dietary groups: an HFHS diet (58% kcal fat and sucrose; D12331, Research Diets, New Brunswick, NJ, USA) or a standard control diet (STD; 11% kcal fat and cornstarch; D12328, Research Diets). Both chow and specialized diets, along with water, remained continuously available for *ad libitum* consumption. Food and water intake were monitored and recorded throughout the study. Additionally, body weight measurements were obtained weekly.

### AT_2_R Agonist Administration

At 28 weeks of age, a subset of female offspring received systemic treatment with the AT_2_R agonist CGP42112 (CGP; MedChemExpress, Monmouth Junction, NJ, USA) via subcutaneous osmotic minipumps (2ML2, Alzet, Cupertino, CA, USA). CGP was administered continuously at 1 μg/kg/min from weeks 28 to 30. This dosage was established based on previous studies demonstrating its efficacy in lowering BP and reducing hypertension-related complications in rat models [28]. The remaining offspring received saline-filled pumps (VEH) for the same duration. Osmotic mini pumps were implanted subcutaneously following previously described surgical methods [29]. Briefly, under anesthesia, rats were positioned prone on a heating pad. A 4×4 cm dorsal area over the iliac crest was shaved and disinfected. A midline incision extending posteriorly towards the ribs was made, followed by the creation of a subcutaneous pocket using blunt dissection to accommodate the pump. The prefilled pump was inserted with the delivery portal leading, and the incision was closed with wound clips.

### Blood Pressure Measurement

BP was measured non-invasively using a CODA tail-cuff system (Kent Scientific, Litchfield, CT, USA) according to established protocols [9]. Measurements were obtained biweekly prior to CGP/VEH administration and at 3-day intervals post-administration. Rats underwent a two-day acclimation period within the restraint chamber prior to data collection. On measurement days, rats were placed in a warmed restrainer, and a 10-minute resting period was allowed to optimize blood flow to the tail. An occlusion cuff and volume pressure-recording cuff were positioned at the base of the tail, undergoing a 90-second cycle of inflation and deflation. Tail pulsation return was recorded. Each rat’s mean BP was calculated by averaging six consecutive readings after discarding the initial five acclimation cycles.

### Heart Weight and Histological Analyses

At 30 weeks of age, female offspring were euthanized via CO_2_ inhalation. Hearts were harvested *en bloc*, dissected to separate atria from ventricles, and weighed individually. Tibia length was measured to normalize the heart weight data. For subsequent processing, hearts were then fixed in 10% formalin (ThermoFisher Scientific, Newark, DE, USA). Following fixation, tissues were paraffin-embedded, sectioned at 4 μm thickness, and mounted onto glass slides. Hematoxylin and eosin (H&E) staining was performed for histological evaluation. Digital image acquisition occurred using an Olympus SZX10 microscope (Olympus Life Science, Tokyo, Japan) equipped with a Q-Color5 imaging system (Olympus Life Science). Morphometric analysis was conducted on captured images employing ImageJ software (version 1.47t). Left ventricular wall thickness was quantified by dividing the left ventricle into four equal sections (anterior, posterior, lateral, and septum). The average thickness of each section, measured from the endocardium to the epicardium, was calculated to represent left ventricular wall thickness.

### Ex-Vivo Vascular Reactivity Assessment

The mesenteric arcade was excised and placed in oxygenated Krebs buffer solution (composition in mM: NaCl 119, KCl 4.7, MgSO_4_·7H_2_O 1.17, KH_2_PO_4_ 1.18, NaHCO_3_ 25, CaCl_2_ 2.5, EDTA 0.03, D-glucose 5.5; pH 7.4). Mesenteric arteries were dissected under a microscope, removing surrounding connective and adipose tissue. Endothelial denudation, when required, was performed by gentle internal abrasion with tungsten wire. Arterial segments (2 mm each) were mounted onto a wire myograph (Danish Myo Techniques, Aarhus, Denmark) for isometric tension measurement. Vessels were equilibrated in Krebs buffer (37°C, aerated with 95% O_2_/5% CO_2_) for 1 hour under resting tension. Optimal resting tension was determined using Myodata normalization software (V8.1.13), setting an internal diameter of 0.9 * L13.3kPa. Endothelial integrity was confirmed by acetylcholine (ACh)-induced relaxation following phenylephrine (PE) precontraction.

After PE-induced precontraction, vascular relaxation responses were assessed in endothelium-intact rings using incremental ACh doses (10^−9^ – 10^−5^ mol/L). Endothelium-independent relaxation was determined by responses to the NO donor sodium nitroprusside (SNP; 10^−9^ – 10^−6^ mol/L) in PE-precontracted endothelium-denuded vessels. The PE concentration eliciting 80% of maximal response (pEC80) was used for precontraction.

### ELISA Assays

Following blood collection, plasma was isolated via centrifugation of EDTA-treated blood samples at 3000 rpm for 15 minutes at 4°C. Supernatants were then collected for subsequent analyses. Enzyme-linked immunosorbent assay (ELISA) kits were employed to quantify plasma levels of Angiotensin II (Ang II; ADI-900–204, Enzo Life Sciences, Farmingdale, NY, USA), bradykinin (EIA-BRAK, RayBiotech, Peachtree Corners, GA, USA), and nitrate/nitrite (NO_X_; 780001, Cayman Chemical, Ann Arbor, MI, USA), following the manufacturer’s protocols. The respective detection ranges for these assays are Ang II (3.9–10,000 pg/mL), bradykinin (0.1–1,000 ng/mL), and NO_X_ (0–35 μM).

### Western Blotting

Mesenteric arteries were homogenized in Radioimmunoprecipitation Assay (RIPA) buffer (Cell Signaling Technology, Danvers, MA, USA) supplemented with a Halt Protease and Phosphatase Inhibitor Cocktail (Thermo Scientific, Waltham, MA, USA) to generate tissue lysates. Following centrifugation, lysates were quantified using a BCA Protein Assay Kit (Pierce, Thermo Scientific). Twenty micrograms of protein were loaded per well onto 4%–12% gradient NuPAGE Bis-Tris gels (Invitrogen, Thermo Scientific) for electrophoresis at 100 V for 2 hours at room temperature. Proteins were then transferred to Immobilon-FL PVDF membranes (MilliporeSigma, Burlington, MA, USA) at 20 V for 1 hour. Membranes were blocked with 5% non-fat dry milk in Tris-buffered saline with Tween 20 (TBST) for 1 hour at room temperature, followed by overnight incubation at 4°C with primary antibodies diluted in TBST with 5% bovine serum albumin (BSA): endothelial nitric oxide synthase (eNOS; 1:1000; #32027, Cell Signaling Technology), angiotensin II type 1 receptor (AT_1_R; 1:1000; AAR-011, Alomone Labs), angiotensin II type 2 receptor (AT_2_R; 1:500; ab92445, Abcam), bradykinin receptor type 1 (B_1_R; 1:500; ABR-011, Alomone Labs), bradykinin receptor type 2 (B_2_R; 1:500; ABR-012, Alomone Labs), and β-actin (loading control; 1:5000; #4970, Cell Signaling Technology). Secondary antibody incubation with IRDye-conjugated probes (IRDye^®^ 800CW and IRDye^®^ 680RD; LI-COR Biosciences, Lincoln, NE, USA) diluted in TBST with 5% BSA was performed for 1 hour at room temperature. Immunoblots were visualized using an Odyssey^®^ XF Imaging System (LI-COR Biosciences), and band intensities were quantified using Empiria Studio software (LI-COR Biosciences). Protein expression levels were normalized to β-actin and expressed as a ratio.

### Statistical Analysis

Statistical analyses were performed using GraphPad Prism (GraphPad Software, San Diego, CA, USA). For comparisons between groups, ANOVA with Dunntet’s post hoc for multiple comparisons was used. For two groups comparisons t-test was used. Vascular reactivity data were analyzed via a four-parameter sigmoidal dose-response curve fitting approach. Relaxant responses to ACh and SNP were calculated as a percentage relaxation relative to PE-induced precontraction. Key parameters, including maximal response (E_max_) and concentration eliciting 50% of the maximal response (pD_2_), were derived from the curve-fitting analysis. Results were presented as mean ± standard error of the mean (SEM) and considered statistically significant at *p* < 0.05.

## Results

### Body Weight and Feed Intake

**Figure 1:**
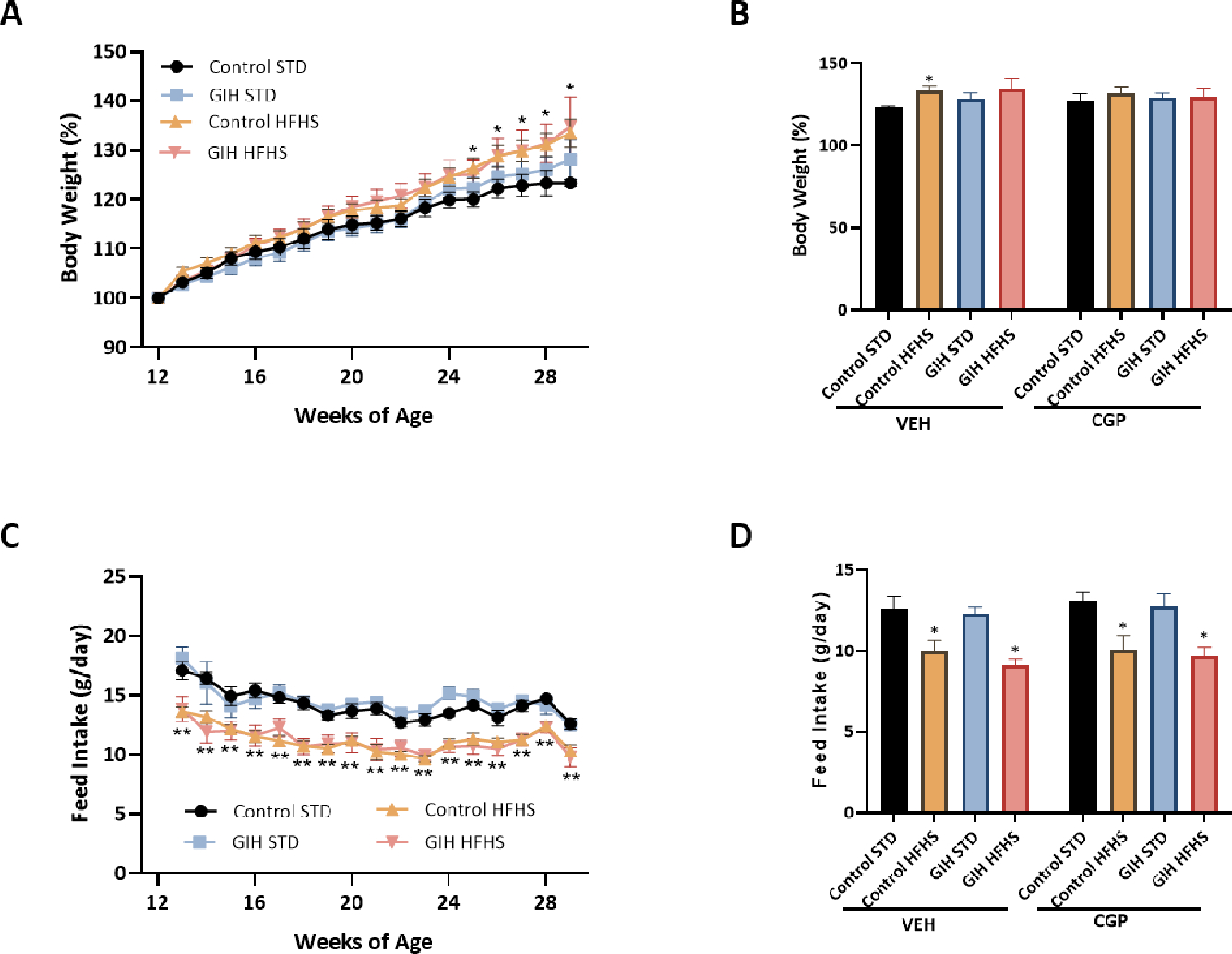
Body weight and feed intake. Female offspring rats exposed to gestational normoxia (Control) or intermittent hypoxia (GIH) exposures were randomly assigned to a standard diet (STD) or a high-fat high-sucrose diet (HFHS) starting 12 weeks of age. Both control- and GIH-exposed female offspring rats with or without HFHS were treated with saline (VEH) or CGP42112 (CGP) from 28 to 30 weeks of age. **(A)** Percent body weight increase measured on a bi-weekly basis. **(B)** Percent body weight change at 30 weeks with and without CGP treatment. **(C)** Average daily feed intake measured on a bi-weekly basis. **(D)** Average daily feed intake with and without CGP treatment. Data are presented as means ± SEM of 12 rats per group before, and 6 rats per group after VEH or CGP treatment. *P<0.05 HFHS vs. STD counterparts.

Birth weight was significantly reduced in female GIH offspring compared with female control offspring (5.9 ± 0.19 vs. 6.7 ± 0.12 g; GIH vs. control, respectively). At 12 weeks of age and throughout the study period of up to 30 weeks of age, body weights did not differ between GIH-STD and control-STD offspring. HFHS feeding initiated at 12 weeks of age led to a significant increase in body weight in control-HFHS but not GIH-HFHS offspring relative to their STD counterparts (Figure 1A). The AT_2_R agonist CGP initiated at 28 weeks of age did not alter the percent change in body weights in all groups relative to their VEH counterparts (Figure 1B). The average daily feed intake was not significantly different between control-STD and GIH-STD offspring (Figure 1C). Similarly, the average feed intake was comparable in control-HFHS and GIH-HFHS offspring up to 30 weeks of age. However, the average feed intake in control-HFHS and GIH-HFHS offspring was significantly lower than their respective STD counterparts (Figure 1C). CGP treatment did not alter daily average feed intake in all groups relative to their VEH counterparts (Figure 1D).

### Blood Pressure

**Figure 2:**
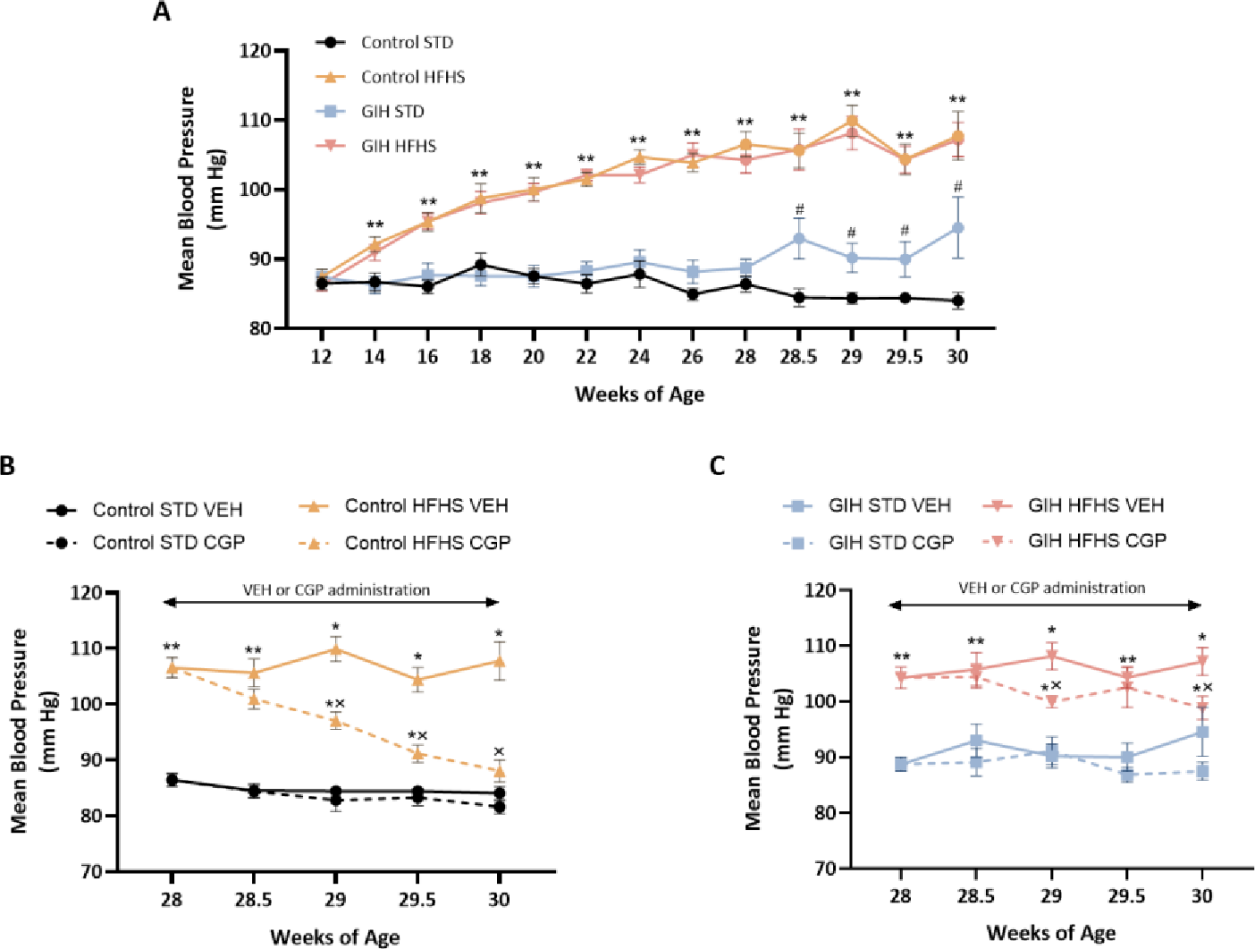
Effect of HFHS and AT_2_R agonist CGP421112 treatment on blood pressure. Mean arterial blood pressure was measured non-invasively using the CODA system. **(A)** Mean arterial blood pressure measured on a bi-weekly basis. **(B)** Mean arterial blood pressure measured after VEH or CGP treatment in control offspring. **(C)** Mean arterial blood pressure measured after VEH or CGP treatment in GIH offspring. Data are presented as means ± SEM of 12 rats per group before, and 6 rats per group after VEH or CGP treatment. ^#^*P*<0.05 GIH vs. control counterparts; *P<0.05 HFHS vs. STD counterparts; ^x^*P*<0.05 CGP vs. VEH counterparts.

Mean arterial BP did not differ between adult control and GIH offspring at 12 weeks of age (baseline). The BP was maintained at similar levels in both control-STD and GIH-STD offspring up to 28 weeks of age, and after that, the GIH-STD offspring exhibited a significantly elevated BP compared to control-STD at 30 weeks of age (Figure 2A). HFHS feeding induced hypertension in both control-HFHS and GIH-HFHS offspring, but the magnitude of BP increase at 30 weeks of age was greater in the control-HFHS offspring (mean increase of 24 mmHg vs. control-STD) than in GIH-HFHS offspring (mean increase of 13 mmHg vs. GIH-STD) (Figure 2A). The AT_2_R agonist CGP initiated at 28 weeks of age decreased BP in control-HFHS offspring and GIH-HFHS offspring; however, at 30 weeks of age, the BP decrease to AT_2_R activation was greater in control-HFHS offspring (mean decrease of 20 mmHg vs. control-HFHS-VEH) (Figure 2B) as compared to GIH-HFHS offspring (mean decrease of 8 mmHg vs. GIH-HFHS-VEH) (Figure 2C). AT_2_R activation did not significantly alter BP in control-STD (mean decrease of 2 mmHg) (Figure 2B) and GIH-STD offspring (mean decrease of 7 mmHg) as compared to VEH counterparts at 30 weeks (Figure 2C). Thus, the reduction in BP response to CGP treatment was greater in control offspring on HFHS diet than in GIH offspring on HFHS diet, suggesting that HFHS feeding led to a differential BP response to AT_2_R activation in female GIH offspring.

### Heart Weight and Structure

**Figure 3:**
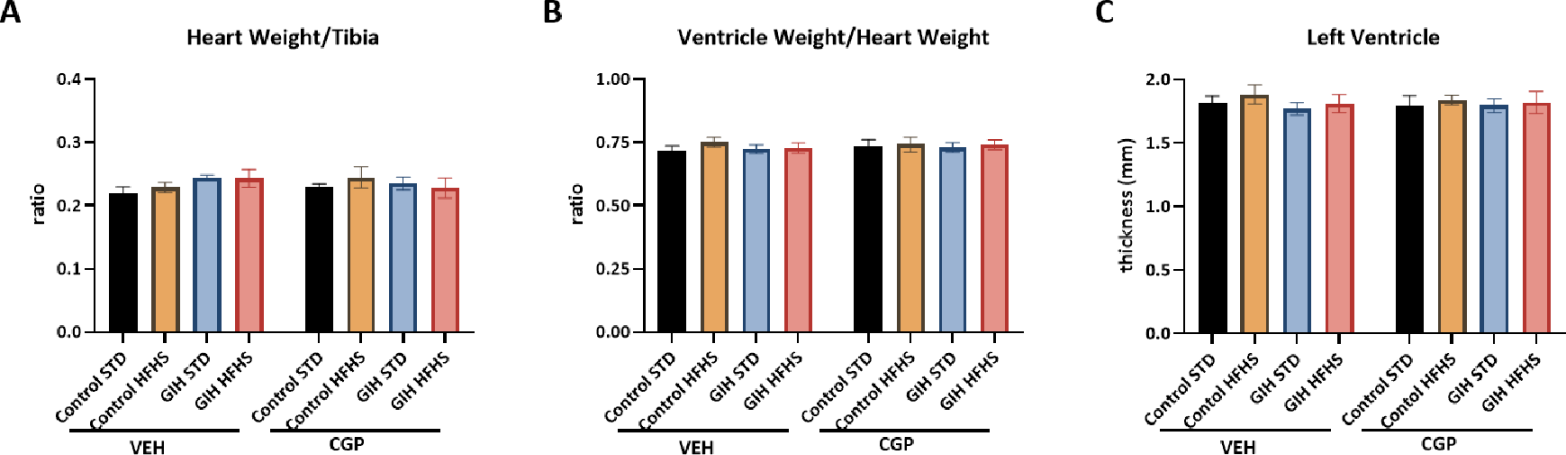
Effect of HFHS and AT_2_R agonist CGP421112 treatment on heart structure. **(A)** Heart weight normalized to tibia length, and **(B)** ventricle weight normalized to whole heart weight were measured. **(C)** Left ventricular wall thickness was analyzed with H&E staining. Data are presented as means ± SEM of 6 rats per group.

GIH exposure, HFHS diet, or CGP treatment did not significantly alter heart weight (normalized to tibia length), ventricle weight (normalized to heart weight), or left ventricular wall thickness (Figure 3).

### Vasodilator Response

**Figure 4:**
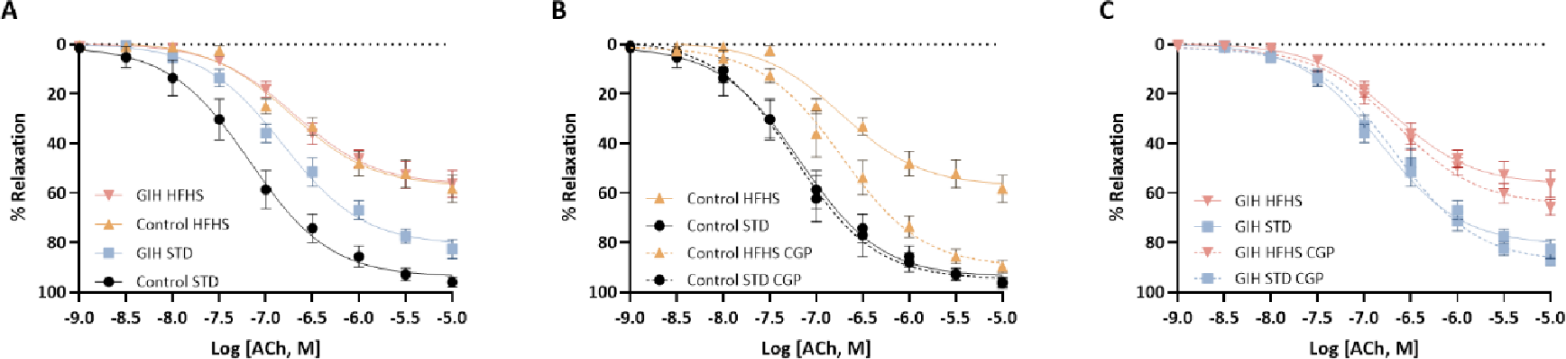
Effect of HFHS and AT_2_R agonist CGP421112 treatment on endothelium-dependent vascular relaxation responses. At 30 weeks of age, mesenteric artery rings isolated from control- and GIH-exposed female offspring, with or without HFHS and CGP treatment, were precontracted with submaximal phenylephrine. The relaxation responses to incremental doses of acetylcholine (ACh) were examined. Data are presented as means ± SEM of 6 rats per group.

ACh-induced relaxation was significantly reduced in endothelium-intact mesenteric arteries with decreased ACh sensitivity and maximal response in GIH-STD offspring than in control-STD offspring (Figure 4A and Table 1). HFHS feeding significantly decreased ACh relaxation responses in both control-HFHS and GIH-HFHS offspring relative to their STD counterparts (Figure 4A and Table 1). CGP treatment restored the decreased ACh relaxation in control-HFHS offspring by increasing ACh sensitivity and maximal relaxation (Figure 4B and Table 1) but did not alter ACh responses in GIH-HFHS offspring as compared to their VEH counterparts (Figure 4C and Table 1). CGP treatment did not significantly alter ACh-induced relaxation responses in control-STD (Figure 4B and Table 1) and GIH-STD offspring as compared to their VEH counterparts (Figure 4C and Table 1).

**Figure 5:**
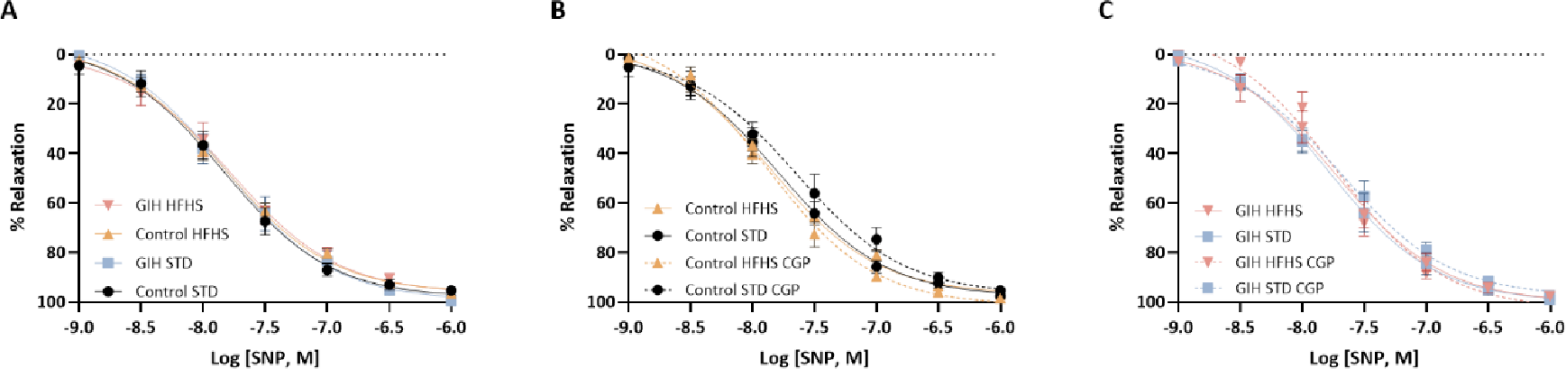
Effect of HFHS and AT_2_R agonist CGP421112 treatment on endothelium-independent vascular relaxation responses. Endothelium-denuded mesenteric artery rings were precontracted with submaximal phenylephrine, and subsequently challenged with incremental doses of sodium nitroprusside (SNP). Data are presented as means ± SEM of 6 rats per group.

SNP, a NO donor, induced concentration-dependent relaxation did not differ upon comparison of all groups (Control and GIH, with or without HFHS diet and CGP treatment) (Figure 5A, B and C and Table 2).

### Circulating Levels of Ang II, NO_X_, and Bradykinin

**Figure 6:**
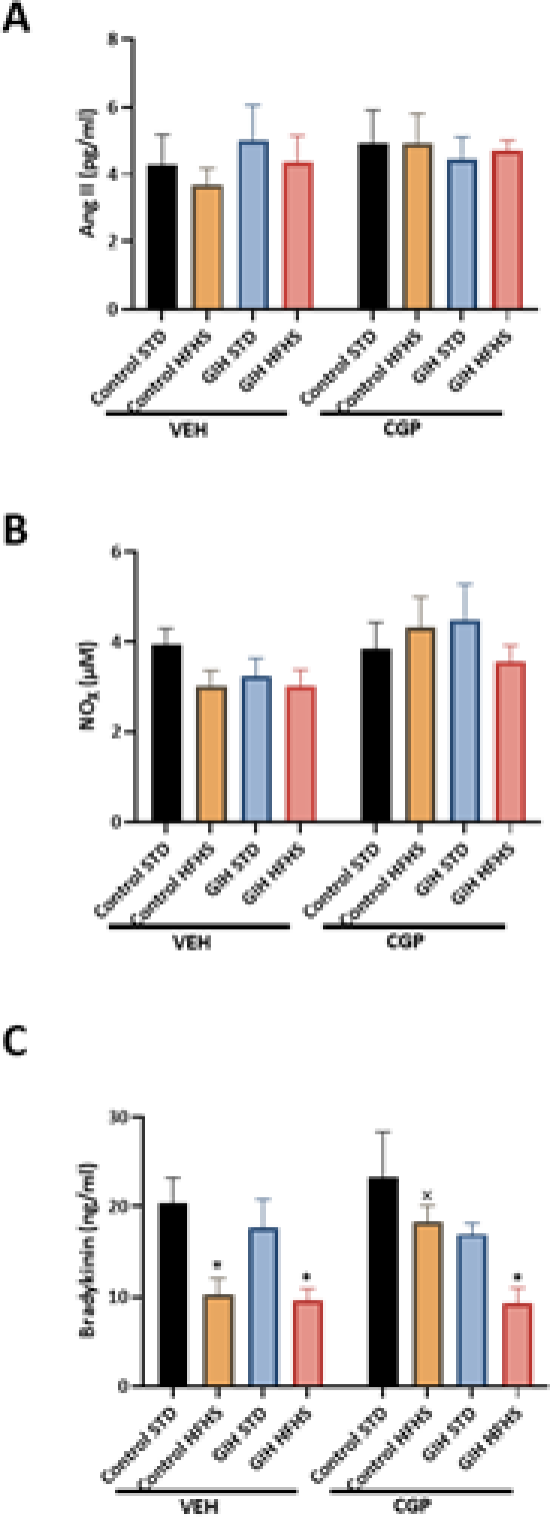
Effect of HFHS and AT_2_R agonist CGP421112 treatment on plasma angiotensin II (Ang II), nitrate/nitrite (NO_X_), and bradykinin levels. At 30 weeks of age, blood was collected from control- and GIH-exposed female offspring rats via cardiac puncture. **(A)** Ang II, **(B)** NO_X_, and **(C)** bradykinin levels were measured using ELISA kits. Data are presented as means ± SEM of 6 rats per group. *P<0.05 HFHS vs. STD counterparts.

Plasma levels of Ang II (Figure 6A) and NO_X_ (Figure 6B) were unchanged across experimental groups.

As shown in Figure 6C, GIH-STD offspring had no significant difference in plasma bradykinin levels compared to control-STD. HFHS feeding led to significantly decreased plasma bradykinin levels in control-HFHS and GIH-HFHS offspring relative to their STD counterparts (Figure 6C). CGP treatment ameliorated the HFHS-induced decline in bradykinin levels in control-HFHS but not in GIH-HFHS offspring as compared to their VEH counterparts (Figure 6C). CGP treatment did not significantly alter bradykinin levels in control-STD and GIH-STD offspring relative to their VEH counterparts (Figure 6C).

### Ang II and Bradykinin Receptors in Mesenteric Arteries

**Figure 7:**
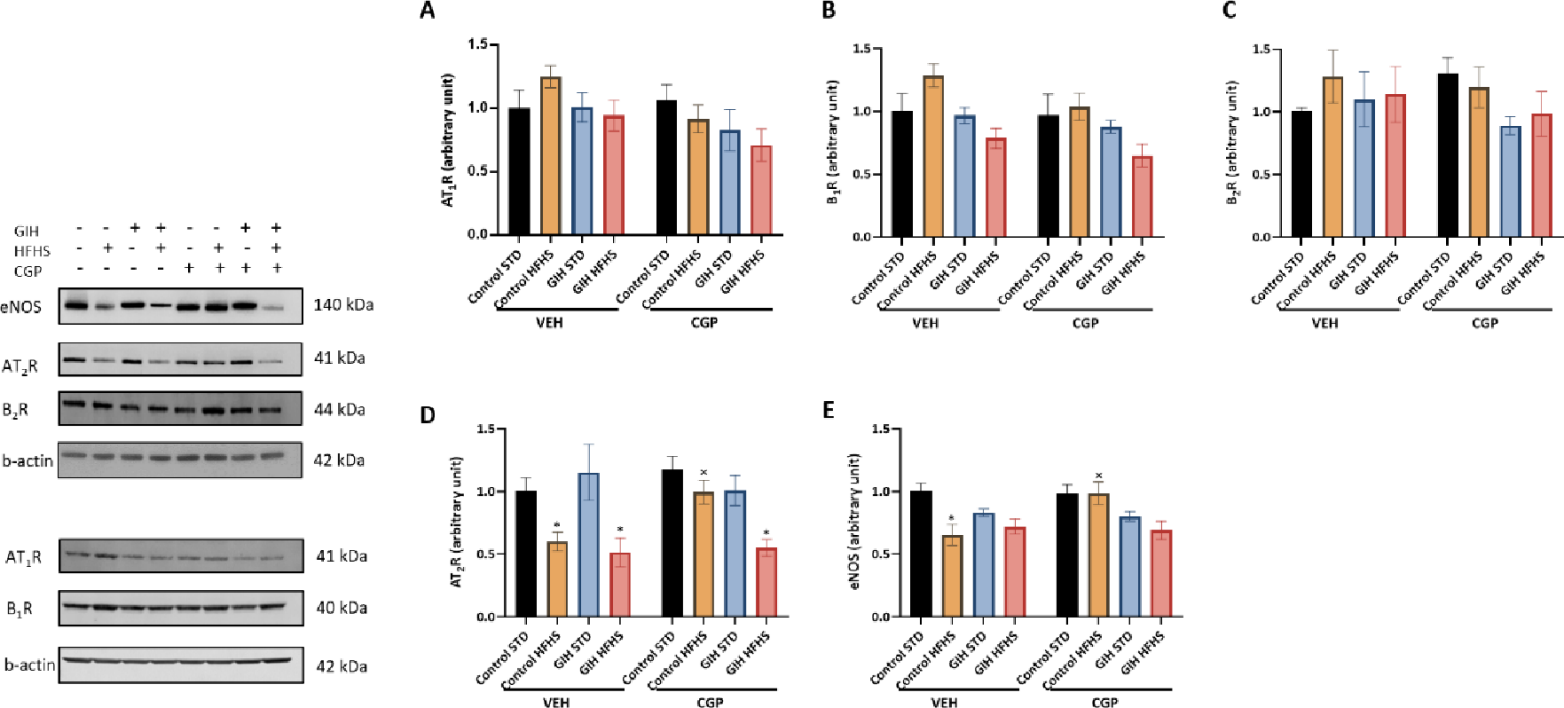
Effect of HFHS and AT_2_R agonist CGP421112 treatment on protein expression of angiotensin II receptors, eNOS, and bradykinin receptors in the mesenteric arteries. Protein expression of **(A)** AT_1_R, **(B)** B_1_R, **(C)** B_2_R, **(D)** AT_2_R, and **(E)** eNOS, in week 30 mesenteric artery samples from control- and GIH-exposed female offspring rats were analyzed using Western blotting. The left panel displays representative bands, while the right panel shows normalized densitometry data. Data are presented as means ± SEM of 6 rats per group. *P<0.05 HFHS vs. STD counterparts; ^x^*P*<0.05 CGP vs. VEH counterparts.

AT_1_R (Fig 7A), B_1_R (Fig 7B), and B_2_R expression (Fig 7C) in mesenteric arteries were similar in all groups.

AT_2_R expression was similar between control-STD and GIH-STD offspring (Fig 7D). HFHS feeding significantly decreased AT_2_R expression in control-HFHS and GIH-HFHS offspring relative to their STD counterparts (Fig 7D). CGP treatment increased AT_2_R expression in control-HFHS offspring but did not affect the GIH-HFHS offspring relative to their VEH counterparts (Fig 7D). CGP treatment did not alter AT_2_R expression in control-STD and GIH-STD offspring relative to their VEH counterparts (Fig 7D). eNOS expression was not altered in GIH-STD compared to control-STD offspring (Fig 7E). HFHS feeding decreased eNOS expression in control-HFHS but not GIH-HFHS offspring relative to their STD counterparts (Fig 7E). CGP treatment prevented the eNOS decrease observed in control-HFHS offspring but did not affect GIH-HFHS offspring relative to their VEH counterparts (Fig 7E). CGP treatment did not alter eNOS expression in control-STD and GIH-STD offspring relative to their VEH counterparts (Fig 7E).

## Discussion

The present study aimed to evaluate the impacts of HFHS feeding as a “second hit” on the GIH-exposed female offspring, a group previously found to be “protective” against maternal intermittent hypoxia exposure-induced cardiovascular dysfunctions. Our key findings are: **(1)** Both control and GIH offspring displayed similar increases in body weight and BP upon HFHS feeding. **(2)** HFHS-induced increase in BP was associated with attenuated endothelial-dependent vascular relaxation, reduced AT_2_R and eNOS expression, and decreased bradykinin levels, while the heart structure and expression levels of AT_1_R and bradykinin receptors remained unaffected. **(3)** Administration of CGP42112, an AT_2_R agonist, effectively reversed HFHS-induced hypertension and endothelial dysfunction in control offspring but not in GIH offspring. **(4)** CGP’s beneficial effects in controls were associated with restored AT_2_R and eNOS expression in the mesenteric artery and increased plasma bradykinin levels; such observations were absent in GIH counterparts. These findings suggest that GIH exposure programs endothelial dysfunction and impairs responsiveness to AT_2_R stimulation during HFHS-induced hypertension in female offspring.

GIH exposure has been associated with the development of preeclampsia-like features during pregnancy, including maternal hypertension, endothelial dysfunction, and fetal growth restriction [30, 31]. Offspring of both sexes from GIH-exposed mothers exhibit catch-up growth after birth [9]; however, the long-term metabolic and systemic consequences appear to be sex-specific [5, 10, 25, 32]. Male offspring exposed to GIH in utero demonstrate negative health outcomes such as obesity, altered blood insulin and lipid profiles, and epigenetic changes in white adipose tissue [33]. Conversely, female offspring from GIH mothers show no significant difference in adiposity, fasting insulin, or lipid profiles compared to controls [33]. Our current study confirms these findings, demonstrating similar body weights in female GIH offspring compared to controls.

We observed less feed intake in the HFHS groups compared to the STD groups, regardless of GIH exposure. This is possibly due to the more energy-dense HFHS diet leading to earlier satiety [34]. However, the total calorie intake remained significantly higher in the HFHS groups, contributing to more pronounced weight gain. Interestingly, HFHS feeding resulted in a significantly higher percentage of weight gain in control offspring compared to GIH offspring. The underlying mechanisms for this resistance to HFHS-induced weight gain in GIH offspring remain unclear. Further investigations are warranted to explore potential differences in energy expenditure, metabolic pathways, and adipose tissue distribution between the groups. In the present study, CGP administration did not alter body weight gain, which is consistent with an earlier observation [35].

Previous studies have demonstrated sex-specific hypertensive consequences of GIH exposure in offspring. Male offspring born to GIH-exposed mothers exhibit hypertension linked to endothelial dysfunction and reduced eNOS activity, whereas females maintain normal BP and endothelial function [9]. This sexual dimorphism might be attributed to the protective effects of functional ovaries in post-pubertal females [11]. Our study observed a rise in BP in GIH female offspring compared to controls at 28 weeks, accompanied by decreased ACh sensitivity in mesenteric arteries. Considering the onset of reproductive senescence in rats around 36 weeks and the established role of ovarian function in BP regulation, particularly during the postmenopausal stage [36–38], these findings suggest a potential adverse impact of GIH on offspring ovarian function and early-onset ovarian aging. This notion aligns with previous studies demonstrating that fetal growth restriction during the critical window of ovarian development (early postnatal period) is associated with reduced ovarian follicle number and impaired follicle growth in post-pubertal guinea pigs [39]. Since maternal GIH is known to induce fetal growth restriction [9, 31], further investigation is warranted to determine whether GIH exposure programs accelerated aging of the female reproductive tract in the offspring.

Interestingly, both control and GIH female offspring exhibited similarly elevated BP upon exposure to the HFHS diet. This finding aligns with observations from the reduced uterine perfusion pressure (RUPP) preeclampsia model, where an HFHS diet induced comparable BP increases in both growth-restricted and sham-operated offspring [40]. However, our study revealed a differential response to the AT_2_R agonist, CGP42112, between control and GIH offspring. CGP42112 administration has been consistently shown to exert protective effects against cardiovascular diseases, particularly hypertension, in various conditions such as atherosclerosis [41], cardiac fibrosis [42], obesity [28], and diabetes [43]. Notably, while CGP treatment reversed HFHS-induced hypertension in control offspring, this beneficial effect was attenuated in GIH offspring.

The mechanisms underlying HFHS-induced hypertension can involve alterations in either cardiac or vascular structure and function. In our study, heart and ventricle weights were comparable across all groups, and H&E staining revealed no evidence of left ventricle hypertrophy following HFHS diet exposure. These findings are consistent with previous research in male mice, where HFHS led to obesity and increased heart weight, but not necessarily in female rodents [44]. Conversely, studies in male and female SD rats subjected to HFHS diets reported no compromise in cardiovascular function [45, 46]. In contrast, in rat experiments, HFHS did not compromise cardiovascular function in SD male or female rats [47]. Similarly, a high-fat diet with normal sucrose levels did not induce cardiac hypertrophy in female rats [48]. Collectively, these observations suggest that in our model, HFHS-induced hypertension is primarily associated with peripheral vascular dysfunction rather than direct cardiac structural changes. However, the potential impact of HFHS on cardiac function cannot be entirely excluded and warrants further investigation.

We examined mesenteric artery function to elucidate the potential vascular mechanisms underlying the observed BP changes with HFHS diet and CGP treatment in female offspring. Various studies have implicated enhanced AT_1_R function and reduced AT_2_R activity within the RAS in diet-induced hypertension [20–22]. Interestingly, our study did not detect significant changes in mesenteric artery AT_1_R protein levels. These findings align with reports showing unaltered AT_1_R mRNA levels in adipose tissue after an HFHS diet [49]. However, they contradict some studies demonstrating increased AT_1_R expression in the kidney, aorta, and adipose tissue following HFHS exposure [20, 21]. These discrepancies might be attributed to variations in species (rats vs. mice) and the specific tissues analyzed.

Our study demonstrates that the elevated BP observed in female offspring fed an HFHS diet was accompanied by impaired endothelium-dependent vasorelaxation to ACh in mesenteric arteries of both control and GIH groups. This finding aligns with previous reports of endothelial dysfunction induced by HFHS diets [50]. Notably, the response to the NO donor SNP remained unchanged between control and GIH offspring, suggesting preserved smooth muscle vasodilatory capacity. Interestingly, CGP administration in HFHS-fed control offspring effectively reversed hypertension and restored endothelial relaxation to levels comparable to controls on a STD. This observation suggests a potential role for AT_2_R activation in enhancing NO synthesis by endothelial cells. Supporting this hypothesis, we observed elevated protein levels of both AT_2_R and eNOS in the mesenteric arteries of CGP-treated controls on the HFHS diet. These findings are consistent with prior studies in various animal models, where CGP treatment was associated with increased AT_2_R and eNOS expression, eNOS phosphorylation, and NO production [41, 43, 51–53]. The precise mechanism by which CGP stimulates eNOS expression remains unclear. However, previous studies suggest a potential involvement of the MEK pathway in type-2 diabetic mice [43] and the PKA/p-eNOS and AKT/p-eNOS signaling pathways in a mouse model of diet-induced obesity treated with Compound 21, a selective AT_2_R agonist [54].

To further explore the impact of HFHS diet and CGP treatment on endothelial function, we measured plasma levels of NO_X_ and bradykinin, key endothelial vasodilators. While no significant changes were detected in NO_X_ levels, this might “reflect substantial changes in systemic NOx rather than localized NO production within the endothelium”,, as dietary sources contribute significantly to circulating NO_X_ levels [55]. Interestingly, we observed significant differences in plasma bradykinin. Bradykinin induces vasodilation via B_2_R binding, leading to increased production of NO, prostacyclin, and endothelium-derived hyperpolarizing factors [56, 57]. These molecules act in a paracrine manner, promoting eNOS upregulation in neighboring endothelial cells. Additionally, B_2_R and AT_2_R can form functional heterodimers, further enhancing NO and cGMP production [58]. The HFHS diet reduced bradykinin levels in both control and GIH offspring, with no apparent effect on B_2_R levels. The underlying mechanism for this reduction requires further investigation. Importantly, CGP treatment in HFHS-fed controls restored AT_2_R function and eNOS expression, coinciding with increased plasma bradykinin levels. This suggests that AT_2_R stimulation enhances bradykinin production, aligning with previous findings [24, 54, 59]. These observations raise the possibility that the improved vasodilation observed in CGP-treated controls on the HFHS diet might be partly mediated by increased bradykinin levels.

Interestingly, CGP treatment failed to restore endothelial function, AT_2_R and eNOS expression or elevate bradykinin levels in GIH offspring on the HFHS diet. Notably, CGP also lacked significant effects in STD-fed animals. This lack of response in GIH offspring aligns with observations in a Zucker rat model, where CGP elicited natriuretic and anti-inflammatory responses only in obese (but not lean) animals [60]. Further studies revealed distinct signaling pathways: CGP treatment only increased cGMP and NO accumulation in obese rats [61]. One hypothesis suggests that differences in AT_2_R’s helix VIII domain might be responsible. This domain affects the receptor’s active state and can hinder the recruitment of G proteins and other signaling molecules [62]. Consequently, AT_2_Rs with specific helix VIII variations may resist desensitization/internalization upon chronic agonist exposure, leading to contrasting functional responses [63]. Previous studies have shown GIH-induced epigenetic modifications in perivascular adiponectin [10]. It is, therefore, worth investigating whether similar epigenetic alterations might affect AT_2_R function and contribute to the differential responsiveness to CGP observed in GIH offspring.

The ineffectiveness of CGP treatment in GIH offspring might also be linked to altered ovarian function. Extensive research demonstrates the protective role of ovaries in BP regulation, particularly in ovariectomized and aging animals [11, 64, 65]. Studies suggest a positive feedback loop between estrogen levels, its receptors, and AT_2_R expression [66–68]. In an Ang II-induced hypertension mouse model, AT_2_R-mediated BP protection was observed only in females, implying ovarian involvement [69]. Maternal environment is known to influence offspring reproductive function [70, 71]. Chronic gestational hypoxia, for instance, accelerates ovarian aging in offspring rats [72]. Similarly, maternal circadian disruption can impair ovarian function in female offspring [73]. It is plausible that female offspring born to GIH-exposed mothers experience early-onset ovarian dysfunction, which subsequently disrupts AT_2_R signaling and endothelial function.

In summary, our study demonstrates that CGP42112, an AT_2_R agonist, effectively reversed hypertension and endothelial dysfunction caused by an HFHS diet in female offspring born to control dams. However, CGP treatment was ineffective in GIH-exposed offspring. These findings suggest that GIH exposure programs long-term cardiovascular dysfunction in female offspring, manifested by altered responsiveness to AT_2_R agonist therapy. This phenomenon may contribute to the clinical challenge of resistant hypertension.

## Figures and Tables

**Table 1:** Vascular relaxation responses to acetylcholine in control- and GIH-exposed female offspring, with and without HFHS and CGP.

	VEH-treated				CGP-treated			
	Control STD	Control HFHS	GIH STD	GIH HFHS	Control STD	Control HFHS	GIH STD	GIH HFHS
**pD**_**2**_	7.13 ± 0.096	5.84 ± 0.106*	6.60 ± 0.082^#^	5.91 ± 0.099*	7.16 ± 0.094	6.64 ± 0.086*^X^	6.55 ± 0.062^#^	6.09 ± 0.073*
**E**_**max**_	95.94 ± 1.93	58.32 ± 5.45*	82.60 ± 4.29^#^	56.28 ± 5.35*	96.44 ± 1.99	89.73 ± 2.46^X^	87.14 ± 2.05^#^	65.69 ± 3.10*

pD_2_ (negative log molar concentration that produces a 50% effect) is presented as −log [mol/L], and E_max_ (maximal responses) is presented as the percent of maximal contraction or relaxation. Results were presented as mean ± standard error of the mean (SEM).

# *P*<0.05 GIH vs. control counterparts;

* *P*<0.05 HFHS vs. STD counterparts;

x *P*<0.05 CGP vs. VEH counterparts.

**Table 2:** Vascular relaxation responses to sodium nitroprusside in control- and GIH-exposed female offspring, with and without HFHS and CGP. Results were presented as mean ± standard error of the mean (SEM).

	VEH-treated				CGP-treated			
	Control STD	Control HFHS	GIH STD	GIH HFHS	Control STD	Control HFHS	GIH STD	GIH HFHS
**pD**_**2**_	7.84 ± 0.085	7.86 ± 0.051	7.79 ± 0.085	7.72 ± 0.082	7.64 ± 0.089	7.89 ± 0.051	7.63 ± 0.085	7.73 ± 0.067
**E**_**max**_	95.36 ± 1.49	96.60 ± 1.56	99.62 ± 2.29	96.88 ± 0.66	100.39 ± 0.80	98.55 ± 0.67	97.39 ± 0.52	98.49 ± 0.58

## Data Availability

All data needed to evaluate the conclusions are present in the paper. Additional data related to this paper may be requested from the author.
